# Reproducing normative femininity: Women’s evaluations of their birth experiences analysed by means of word frequency and thematic analysis

**DOI:** 10.1186/s12884-021-03758-w

**Published:** 2021-04-14

**Authors:** Agneta Westergren, Kerstin Edin, Monica Christianson

**Affiliations:** 1 Department of Nursing, Umeå University, SE-901 87 Umeå, Sweden; 2 The Graduate School of Gender Studies, Umeå University, Umeå, Sweden; 3 Department of Epidemiology and Global Health, Umeå University, Umeå, Sweden

**Keywords:** Childbirth, Parturition, Birth experience, Patient satisfaction, Gender identity, Femininity, Midwifery, Qualitative data analysis

## Abstract

**Background:**

Given the significance of the birth experience on women’s and babies’ well-being, assessing and understanding maternal satisfaction is important for providing optimal care. While previous research has thoroughly reviewed women’s levels of satisfaction with the childbirth experience from a multitude of different angles, there is a dearth of papers that use a gender lens in this area. The aim of this study is to explore through a gender perspective the circumstances attributed to both women’s assessment of a positive birth experience and those which contribute to a lack of satisfaction with their birth experience.

**Methods:**

Through the use of a local birth evaluation form at a Swedish labour ward, 190 women gave written evaluations of their birth experiences. The evaluations were divided into groups of positive, ambiguous, and negative evaluations. By means of a latent and constructionist thematic analysis based on word count, women’s evaluations are discussed as reflections of the underlying sociocultural ideas, assumptions, and ideologies that shape women’s realities.

**Results:**

Three themes were identified: *Grateful women and nurturing midwives doing gender together* demonstrates how a gender-normative behaviour may influence a positive birth experience when based on a reciprocal relationship. *Managing ambiguous feelings by sympathising with the midwife* shows how women’s internalised sense of gender can make women belittle their negative experiences and refrain from delivering criticism. *The midwifery model of relational care impeded by the labour care organisation* describes how the care women receive during labour and birth is regulated by an organisation not always adapted to the benefit of birthing women.

**Conclusions:**

Most women were very satisfied, predominantly with emotional support they received from the midwives. The latent constructionist thematic analysis also elicited women’s mixed feelings towards the birth experience, with the majority of negative experiences directed towards the labour care organisation. Recognising the impact of institutional and medical discourses on childbirth, women’s birth evaluations demonstrate the benefits and challenges of gender-normative behaviour, where women’s internalised sense of gender was found to affect their experiences. A gender perspective may provide a useful tool in unveiling gender-normative complexities surrounding the childbirth experience.

## Introduction

The experience of childbirth is, for many women, a major life event or a liminal rite of passage, with a lifelong impact on her physical, psychological, and social self [1–3]. A positive and enriching birth experience is linked to women’s feelings of accomplishment, heightened self-esteem, and confidence in their ability to face the challenges of motherhood [2, 4, 5]. Conversely, a negative birth experience may have profound immediate and long-term effects, increasing the risk for posttraumatic stress disorder and postpartum depression, which, in turn negatively affects bonding between the mother and the new-born and breastfeeding [3, 6–8]. A negative birth experience is also a predictor for future caesarean section by maternal request and is associated with fewer subsequent children and a longer interval between the first and second child [9, 10].

Given the significance of the birth experience on women’s and babies’ well-being, assessing and understanding maternal satisfaction is important for health care providers, administrators, and policymakers, in order to provide optimal care [11, 12]. However, satisfaction is a complex concept, involving both an affective response to an experience, as well as a cognitive evaluation of that response [11]. Furthermore, evaluation measures may fail to fully identify the very core of satisfaction, as they do not always distinguish between the experience of care and the often emotional experience of labour and birth, and women may be satisfied with some aspects of the experience and dissatisfied with others [12, 13].

A search of the literature showed that maternal satisfaction is indeed multidimensional, involving assessments of personal control, whether expectations are met, perceived pain, and practical and emotional support [1, 12, 14]. Among factors that increase the risk for a negative birth experience are situations or interventions directly associated with the birth event, such as induction and augmentation of labour, epidural analgesia, instrumental delivery, prolonged labour, anal sphincter injury, and emergency caesarean sections [9, 15–18]. Women have also reported dissatisfaction with the physical environment, interpersonal care, information and decision-making, and with experiences involving the admittance of their newborns to a neonatal intensive care unit [16, 19].

Adding to the complexity of measuring maternal satisfaction is that birth in most care contexts often involves interpersonal relationships, as birthing women are assisted by midwives, nurse-midwives, nurses or physicians. A large systematic review found that personal expectations, the amount of support received from caregivers, the quality of relationship between woman and caregiver and her involvement in decision-making, were more important than age, socioeconomic status, ethnicity, childbirth preparation, the physical birth environment, pain, immobility, medical interventions, and continuity of care, in terms of influence on level of satisfaction [11]. Drawing on the review findings, Hodnett [11] contends: ‘The influences of pain, pain relief, and intrapartum medical interventions on subsequent satisfaction are neither as obvious, as direct, nor as powerful as the influences of the attitudes and behaviours of the caregivers.’

It can be argued that the attitudes and the behaviours of the caregivers, as well as of the birthing women, are reflections of the context and culture in which the birth takes place. In high and middle income countries, childbirth is becoming increasingly technological and medicalised, and interventions during birth have become more the rule rather than the exception, at times increasing the risk for both woman and child [20]. Swedish maternal and neonatal outcomes are among the best in the world, but in spite of increasing obstetrical interventions intended to further improve outcomes, maternal and neonatal mortality have remained largely unchanged the past ten years [21].

While previous research has thoroughly reviewed women’s levels of satisfaction with the childbirth experience from a multitude of different angles, there is a dearth of papers that use a gender lens in this area. Globally, there is a positive trend towards feminist midwifery research addressing inequalities in maternity care due to gender, but the impact of gender normative behaviours, or femininity, in connection with labour and birth, and the evaluation thereof, largely remains terra incognita. The aim of this study is thus to explore the circumstances influencing the extent to which women are—or are not—satisfied with their birth experiences, through the perspective of gender normativity.

## Methods

### Gender perspectives on birth experiences

Present work explores the reproduction of normative femininity in the birthing room, based on the concept of gender as a mechanism of social control, transmitted through institutional practices and discourses, and subsequently internalised by individuals, who discipline themselves according to prevailing notions of gender [22]. In line with West and Zimmerman [23], we understand gender as partly imposed by societal norms and arrangements, but also as something women and men actively do, constructing themselves as feminine or masculine (or both or neither), which influences every part of our lives: who we are, how we present ourselves, how we act, how we birth, and also how we evaluate our births.

Beginning from an early age, girls and boys are socialized into conforming to certain norms and expectations based on gender affiliation. This gender-appropriate behaviour becomes so familiar that it can seem to be part of a natural order, predicted by individual biology: ‘women are supposed to be nurturant, suggestible, talkative, emotional, intuitive, and sexually loyal; men are supposed to be aggressive, tough-minded, taciturn, rational, analytic, and promiscuous’ [24]. Recognising that there are multiple ways of expressing gender identity and that there are many forms of femininity, present work understands normative femininity according to Western standards within the framework of Gilligan [25], whereby women are expected to be nice, kind, polite, caring, relational, and selfless.

Sociological and feminist research on childbirth argues that patriarchal ideology and social institutions like the medical system, shape women’s birth experiences and affects their health, in that it promotes a view of pregnancy, labour, and birth as abnormal conditions in need of medical and technological control [26–28]. This process is usually referred to as the medicalisation of childbirth, and according to its critics, it promotes the disciplining of women’s bodies, leading to birthing women’s disempowerment and loss of agency [29].

While the sociological and feminist literature has added to the understanding of how the birth experience is shaped by the macro and institutional contexts surrounding birth, there are few studies on women’s internalised senses of gender which they bring with them to the birthing room. Martin [29], demonstrated how birthing women’s ‘internalized technologies of gender’, or mechanisms through which individuals learn socially appropriate ways of being, restricted their behaviour, made them feel bad about their actions, and made them reluctant to impose on others or express a need for help during birth. Similarly, Carter [22], explored how birthing women prioritised certain aspects of femininity over others, in particular the ‘good mother’.

Midwifery, is a dynamic profession encompassing empathy, intuition, experience, skills, and knowledge, and midwives need to continuously question and evaluate their own practice, and remain aware of current evidence for best practice [30, 31]. Being a relatively new academic discipline, midwifery science draws on the theory and knowledge of other disciplines, for instance psychology and sociology. Given the gendered context of childbirth in Sweden, where most birthing people and 99.7% of the midwives are women [32], a gender perspective may prove a powerful resource in offering a deeper understanding of the forces that shape women’s lives [33]. In this paper, we thus draw on feminist theory to show that women’s birth experiences are ‘socially constructed rather than built directly upon biology or the materiality of the body’ [28]. Taking as a point of departure the gendered context of Swedish childbirth, we hold that the birthing room is an apt arena for the study of how the doing of gender affects both the care given and the care received, ultimately influencing how women evaluate their birth experiences.

### Setting

In Sweden, antenatal, labour, and postnatal care is mainly government funded and universal for all. Almost all women give birth in hospitals, home births are rare, and continuity of care is not an option for most women, except as part of a research project. During labour and birth, most women are attended to by midwives, who, supported by assistant nurses, work independently with normal births [34]. In cases of an emergency or of deviations from the normal process, a physician is summoned, collaborating with the midwife in charge to handle the situation. Even though Swedish midwives are the primary caregivers and responsible for normal labour and birth, the organisation in which they work is led by obstetricians, who are responsible for the development of and adherence to clinical practice guidelines.

This study was conducted at a highly technical and medically advanced Swedish hospital labour ward with an annual birth rate of approximately 2000 births. It builds on previously published research, showing that women are very satisfied with their birth experiences, despite not having their preferences for pain relief met (having more pharmacological pain relief than intended), and despite almost 80% having some form of birth intervention [35].

### Participants

A consecutive sample of four hundred women who gave birth in the aforementioned hospital between March and June 2016, were handed written information about the study a few hours after birth, regardless of their age, parity, ethnicity, mode of delivery, or preferences for pain relief. Of the 400 women, 259 (64.8%) consented to participate. Of these, 190 women (73.4%) gave a written evaluation of their birth experience, described in more detail below. Table 1 shows the characteristics of the participants and the rate of intrapartum interventions as documented in their medical records. Of note, medical records do not inquire as to the participants’ preferred gender identity, assuming they all identify as women. Since there is no way of knowing their preferred gender identity, in this paper we use the terms ‘woman’, ‘she’, and ‘her’ for all participants and midwives alike although this may disagree with the gender and pronoun preferences for some.

**Table 1:** Characteristics of participants (*n* = 190), rate of interventions, and VAS

CHARACTERISTICS	n (%)
**Age**	Mean 30.8 (sd 4.6) (min/max 18–43)
**Parity**	
Primiparas	79 (41.6)
Multiparas	111 (58.4)
**Civil status**	
Cohabiting	187 (98.4)
Live-apart	3 (1.6)
**Geographical background**	
Swedish	168 (88.4)
Non-Swedish	22 (11.6)
**Level of education** ^**a**^	
Higher	106 (55.8)
Primary or secondary	64 (33.7)
Not specified ^b^	20 (10.5)
**INTRAPARTUM INTERVENTIONS**	
Induction of labour ^c^	33 (18.6)
Augmentation of labour ^c^	79 (44.6)
Amniotomy ^c^	78 (44.1)
Epidural analgesia ^c^	58 (32.8)
Continuous foetal monitoring ^c^	117 (66.1)
Urinary catheterisation ^c^	74 (41.8)
Vacuum-assisted birth ^c, d^	6 (3.7)
Episiotomy ^c, d^	8 (5.0)
Emergency caesarean	16 (9.0)
Elective caesarean	13 (6.8)
Accumulated interventions ^c, e^	138 (78.0)
**VAS**	
VAS 0–10 Birth Experience ^f^	Median 8.3 (min/max 0.3–10.0)

^a^ According to the Swedish Standard Classification of Occupations [36]

^b^ Students, unemployed or on parental leave

^c^ Elective caesareans excluded (*n* = 13)

^d^ Emergency caesarean section excluded (*n* = 16)

^e^ Number and percentage of women having one or more of listed interventions

^f^ 0 = Very negative and 10 = Very positive

To ascertain whether women without written evaluations (*n* = 69) did not avoid rating their experiences because they were negative, a non-response analysis was conducted. Apart from a significant difference in vacuum-assisted birth, which occurred in 13.0% of women without a written evaluation vs 3.2% for women with a written evaluation, there were no differences in characteristics, rate of other intrapartum interventions, or in Visual Analogue Scale (VAS) rates of satisfaction.

### Data collection

An evaluation form designed by labour ward midwives which was already in use as part of this specific labour ward’s quality management system was collected, together with data from women’s medical records. The evaluation form consisted of a single sheet of paper measuring satisfaction with the birth experience using VAS, offering counselling within three months postpartum to the women who rated their experience as a three or lower. Along with the VAS there was also a text encouraging the women to write something about their experience on five dotted lines: ‘For you who just became a mother. Congratulations! We who work with health care development and postpartum follow-up at the delivery ward would like to know how you experienced your birth. What do you feel when you think about your birth? What do you think was good? What could have been done better?’

The form was handed to the woman a few hours after birth by the midwife who assisted her during birth, and later collected by another midwife at the postpartum ward. Most women filled in the form prior to being discharged from the hospital, generally within 48 h after birth. Almost all wrote one to three sentences, but a few women were more elaborate in describing their experiences.

### Data analysis

Thematic analysis provides a flexible approach to the interpretation of qualitative data and is an apt method for summarising key features of a large data set. To mitigate the risk that the very same flexibility can lead to inconsistency and lack of coherence in developing themes, applying and making explicit an epistemological position may be useful in the analytical process [37]. Therefore, in addition to the six steps of thematic analysis described by Braun and Clarke [38]: familiarisation with the data; initial coding; searching for themes; reviewing themes; defining and naming themes; and producing the report, we used a gender perspective to inform the analytical process as well as the discussion. Through conducting a constructionist thematic analysis, we sought to theorise the sociocultural context of women’s birth evaluations.

Crushing the myth that qualitative researchers do not count, Sandelowski [39] contends that numbers are in fact integral to qualitative research, as meaning depends, in part, on number. Pattern recognition in data implies identifying recurring patterns, and displaying information numerically can help avoiding over- or underweighting data, or undervaluing the messiness of human accounts and lives [39]. Therefore, in our analysis of women’s birth evaluations, we not only took into consideration what the women wrote but also how many times they wrote it. Basing codes and preliminary themes on the prevalence of certain words and recurring expressions enabled us to gain a deeper insight into what women valued most and what they thought could be improved.

**Fig. 1:**
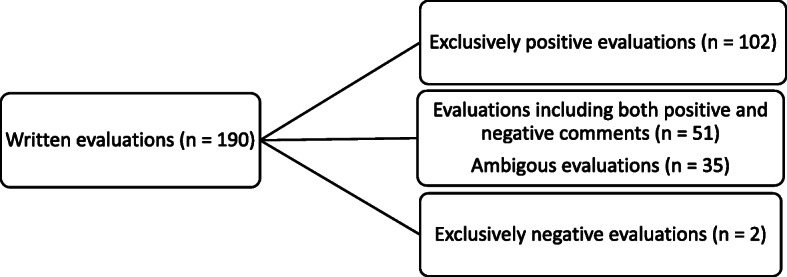
Distribution of evaluations

Familiarization with the data made it clear that most women wrote exclusively positive evaluations. There were also women whose evaluations contained one comment on something they were satisfied with, and one comment on something they thought could have been done better. Forming a category of their own were evaluations where women, often in the same sentence, expressed ambiguous feelings towards the experience, feeling both positive and negative at the same time. Only two women wrote evaluations that were exclusively negative. Reviewing the data also made it clear that some words and expressions were used more frequently than others. In order to not confound positively and negatively charged words during the word counting process, a distinction was made between evaluations that were written in a positive manner, and those that were perceived as less positive. Positive evaluations were identified through the use of adverbs, superlatives, and words describing satisfaction and positive feelings, while negative feelings, critique of care, and ideas for improvement were labelled as negative evaluations. Table 2 illustrates examples of the sorting process for the different types of evaluations and Fig. 1 shows the distribution of evaluations.

**Table 2:** Examples of sorting process of positive, negative, and ambiguous evaluations

Different types of evaluations	Sorting category
*We were both incredibly well received and attended to by staff who were amazingly dedicated, present, and perceptive. I got exactly the support I needed (pain relief, encouragement, tips and advice) when I needed it – many times without even asking for it. Right now, I don’t feel there’s anything I would have wanted differently. Thank you!*	Exclusively positive evaluation
*Good: That I had a quick birth.* *To be improved: I would have wanted to have a midwife present in the labour room the whole time, since I could have started pushing earlier if she had been there.*	The same woman leaving one positive and one negative comment ^a^
*The birth was quite fine really, everybody was nice, the pain relief ok, it was quick and easy, but I still have memories from my last birth fresh in mind (which wasn’t quite as good), which I think colours my experience a little.*	Ambiguous evaluation
*Negative: There was no time for pain relief.*	Exclusively negative evaluation

^a^ During the analysis the first sentence was sorted into positive evaluations, and the second into negative evaluations

#### The coding process

The separated evaluations were run in a word count tool, generating a list of the number of times each word occurred. Basic words without direct meaning, like ‘and, or, but, if’ were omitted, as were words not forming any pattern together with other words, and those not related to the birth evaluation. While this procedure exhibited a clear pattern within the positive evaluations, it did not for the negative or the ambiguous evaluations, which were fewer in number and more disparate, as single words became unintelligible outside their wider context. Thus, the analysis of the positive evaluations is based on recurring words, and the analysis of the negative and the ambiguous evaluations on recurring expressions. To ensure the correct use or intended meaning of the words, the original text was referred to for contextualisation if there was any doubt during the coding process. Tables 3 and 4 show coding examples.

**Table 3:** Example of coding of positive evaluations, based on word frequency

Words (number of times used)	Codes	Preliminary theme
Staff (49), the staff (43), the staff’s (1), operating room staff (1), professions (1), they/you [the staff] 11, midwife (28), the midwife (28), the midwife’s (1), midwives (6), the midwives (4), student midwife (2), doctor (4), physician (1), the anaesthesiologist (1), nurse (1), nurses (3), assistant nurse (1), the assistant nurse (1), assistant nurses (1), the assistant nurses (1)	Staff ^a^ (106), midwife (69), physician (6), nurse (4), assistant nurse (4)	The midwife being central to the woman’s birth experience

^a^ Birthing women, or patients in general, may not always be aware of the job title of the caregiver, offering an explanation to why many of them wrote ‘staff’ instead of ‘midwife’. As there were no nurses present in this specific labour ward we have interpreted ‘staff’ and ‘nurse’ as ‘midwife’ if not otherwise specified

**Table 4:** Example of coding of negative and ambiguous evaluations, based on recurring expressions

Expression	Codes	Preliminary themes
*[A] prolonged latent phase with very drawn-out, constant pain made the first days a horrible experience. I didn’t receive the support I needed and it felt like they didn’t understand me. I felt powerless and out of control of the situation. It was dreadful!*	Constant pain Not receiving wanted support Not being understood Feeling powerless and out of control	Pain and discomfort Invalidated and neglected Lack of information and dialogue
*The staff was great but the birth experience was dramatic and frightening. I don’t think anything could have been done better. I feel that I got the help that I needed despite an overcrowded labour ward.*	Great staff but dramatic and frightening Nothing could have been done better Received adequate support despite full ward	Difficult experience but good support

The preliminary themes were assembled and sorted into three overarching themes. Drawing on Braun and Clarke [38], the final themes are based on prevalence but go beyond the semantic content of the data, instead being identified at a latent level. Thus, they describe not only *what* or *how often* the women wrote something, but seek to identify and examine underlying ideas, assumptions, and ideologies as to *why* they wrote it. Table 5 gives an overview of how codes and preliminary themes underpin the final themes.

**Table 5:** Overview of theme development

	Codes	Preliminary themes	Themes
**Positive evaluations**			
**Code frequency (words)**			Grateful women and nurturing midwives doing gender together
87	Very (42), Absolutely (25), Really (9), Extremely (6), Incredibly (5)	Adverb intensifiers emphasising the positive evaluations	
301	Good (201), Great (33), Super (24), Fantastic (18), Awesome (15), Wonderful (7), Amazing (3)	An overall positive birth experience	
189	Staff (106), midwife (69), physician (6), nurse (4), assistant nurse (4)	The midwife being central to the woman’s birth experience	
99	Gratefulness (33), Positive feelings (25), Satisfaction (23)	Women being grateful and satisfied	
229	Receiving/responsive (64), Safe (40), Perceptive (31), Sweet (20), Calm (19), Lovely (10), Caring (8), Compassionate (8), Accommodating (6), Personal (5), Respectful (5), Enthusiastic (4), Warm (4), Attentive (2), Kind (2), Happy (1), Honest (1)	The caring midwife	
115	Supportive (46), Helpful (25), Encouraging (14), Tips (15), Guiding (11), Present (5)	The supportive midwife	
65	Information (22), Pedagogical (19), Communication (16), Inclusive (8)	The informative midwife	
65	Competent (38), Professional (27)	The competent midwife	
**Code frequency (expressions)**			
**Ambiguous evaluations**			
41	Difficult experience but good support (26), Excusing being neglected (11), Not as expected but ok (4)	Not quite satisfied but thankful and understanding	Managing ambiguous feelings by sympathising with the midwife
**Negative evaluations**			
22	Absent midwife (9), Invalidated (7), Badly treated (6)	Invalidated and neglected	The midwifery model of relational care impeded by the labour care organisation
22	Too little information midwife to woman/partner (14), Lack of communication midwife-woman or midwife-other staff (4), Midwife didn’t read the birth plan (4)	Lack of information and dialogue	
20	Critique of care and routines (10), Opinions on physical environment (10)	Critique of care, routines, and physical environment	
10	Pain (4), Epidural too late (4), Negative emotions (2)	Pain and discomfort	

## Results

Based on the positive, ambiguous, and the negative birth evaluations, we identified three themes: *Grateful women and nurturing midwives doing gender together; Managing ambiguous feelings by sympathising with the midwife*; and *The midwifery model of relational care impeded by the labour care organisation*.

### Grateful women and nurturing midwives doing gender together

More than half of the women (*n* = 102) evaluated their birth experiences as exclusively positive, 51 women wrote one positive and one negative comment, 35 women wrote one ambiguous comment, and two women wrote exclusively negative comments. As a whole, women were very satisfied, which is also confirmed by a VAS median of 8.3 (Table 1).

Furthering the analysis as to what aspects of the birth experience women found satisfactory, the most frequently used words were positive adjectives, sometimes in the superlative form or used with adverb intensifiers, either describing the overall positive birth experience or the midwives. Moreover, the word count suggests that the person or persons surrounding and assisting the women during labour and birth were central in achieving a positive evaluation, as many evaluations contained words on staff.

While most women described their birth experiences in a positive light, few of them mentioned their own feelings of accomplishment or empowerment. In fact, only one woman wrote that she felt ‘strong and proud’ after birth. Instead, the women rather placed their focus on the midwives, expressing satisfaction and deep gratitude towards the midwives for the way they made the women feel.

*‘Perfect! :) Nothing could have been done better. I/we had a fantastic experience. [The midwife was] competent, resolute, awesome. I really liked that our midwife stayed along at the change of shift. :) A thousand thanks for a wonderful memory that I will treasure for the rest of my life. Hugs.’*

There were also accounts of women expressing gratitude over the midwife allowing them to ‘act out’ or behave in a certain way, placing themselves in a subordinate position and leaving it up to the midwife to accommodate their wishes or not.

*‘Good. It turned out the way I wanted. Natural. The midwife let my body birth. The position felt natural. Good guidance.’*

Exploring the circumstances to which the majority of women attributed their satisfaction with the midwives, the most often used words were primarily connected to the midwives’ attitudes and attributes that the women seemingly appreciated and held in high regard. Words describing caring traits were used abundantly, such as perceptive, sweet, calm, lovely, compassionate, and accommodating. These nurturing traits are usually associated with a socially constructed norm of femininity, suggesting that this enactment of feminine normativity is valued by the women, demonstrating an appreciation and a need for the so-called soft values in childbirth.

*‘Calm, reassuring, warm, empathetic midwife [name].’*

Another indication of the appreciation of birthing women of characteristics historically, socially, and culturally associated with a gender-appropriate behaviour for western women, such as being relationally oriented and humbly sensitive to the needs of others, is the mentioning of and appreciation for the midwives’ presence and supportiveness. There was a recurrence of words describing how the midwife engaged with the woman/couple, providing both emotional and practical support to assist the woman coping with labour pain.

*‘I feel that I had great support during the whole birth. We had an amazing midwife [name] who was very supportive and present, and gave good tips and advice regarding relaxation, breathing, different birth positions etc.’*

Several women expressed how the midwife supported them to a positive experience and in response they praised not only the midwife’s work but her persona as well. This captures the essence of the midwifery model of care – woman-centred and building on a reciprocal relationship that bridges the patient-care provider gap between woman and midwife.

*‘Midwife [name] is an amazing midwife who really puts the birthing woman at the centre of attention. You definitely feel that she is on your side. She as a person is the sole reason for our positive birth experience.’*

Although midwifery encompasses informative and medical/technical skills as well as emotional and supportive ones, the word count revealed that the former were mentioned half as often. However, a few women expressed being reassured and satisfied with various techno-medical interventions during childbirth, such as the below mention of continuous foetal monitoring.

*‘I really appreciated that the staff paid so much attention to us as they did and that they closely monitored the baby the whole time. Even if the situation was stressful for the baby, I nevertheless felt that I was in good hands.’*

### Managing ambiguous feelings by sympathising with the midwife

There were several evaluations that were ambiguous, describing women’s undetermined feelings towards the birth experience, being satisfied and dissatisfied at the same time. These evaluations expressed two contrasting ideas in the same sentence, often containing words like ‘but’, ‘despite’, ‘even though’ or ‘anyway’. Some of these accounts began with ‘it was hard but…’, listing hard work, severe pain, or loss of control as the reason for a difficult, intense, painful, dramatic, frightening – but yet positive birth experience.

*‘It was hard and exhausting but at the same time an amazing experience.’*

Other ambiguous evaluations began with ‘it was great but…’, the women feeling they had received the support they needed and that their wishes had been met, but still felt overwhelmed negatively by the birth experience, or labour pain, more specifically.

*‘The staff was great and I feel that I received the support that I needed. The actual birth went very well, it was quick and without any problems, and it actually turned out exactly the way I had hoped it would. I had planned to use only nitrous oxide which I did. The reason why I’m not rating the experience any higher [6.4] despite all this, is that giving birth is really very hard!’*

Some women, although perceiving they did not have the support or the birth experience they had hoped for, belittled their own feelings and/or requests, making excuses for the midwives’ absence and showing great understanding for their stressful work situation. Being sympathetic to other women and downplaying criticism is here interpreted as women’s collective experiences of subordination.

*‘Generally, I was treated well. Great encouragement! Calm start, the bath was pure luxury! I was fine with labouring on my own but I remember feeling frustrated when the contractions were augmented with Oxytocin in the morning whereas I wanted more support. But then the delivery ward was full and as everything progressed according to plan for us, it was understandable [that she did not receive desired support]. Great support and guidance during the pushing phase though, when I really needed it.’*

In a similar vein, some women seemed to mitigate their critique of care by simultaneously expressing gratitude for the midwife’s valuable work. Although it may be possible to be critical and grateful at the same time, we interpret this approach as indicative of gender-appropriate behaviour, where women are expected to be nice, patient, and grateful, making it more difficult to have an opinion or to assert one’s rights. The ambiguous evaluations may be an expression of this discrepancy between normative expectations regarding women’s conduct and their actual feelings and experiences.

*‘I thought everything went along smoothly and there was time for me to have the epidural just like I wanted. I (we) felt that the midwives were very occupied the whole night so ‘our’ midwife was gone for long periods of time, but it felt ok anyway. She did a* *very* *good job and managed (despite a heavy workload) to make us feel safe. But of course, I would have preferred to have someone with us more. During my last birth we had a midwifery student (I think) present for the larger part of labour and birth, which was really helpful! If my partner hadn’t asked for the epidural this time I’m worried that I wouldn’t have had one since the midwife was so busy.’*

### The midwifery model of relational care impeded by the labour care organisation

Although not of the same magnitude as the positive evaluations, there were also negative evaluations providing insights to some aspects of care with which women expressed lower levels of satisfaction. Not all were negative but offered suggestions for improving care.

While the impact of the attitudes and behaviours of the midwives were apparent in the positive evaluations, so was the case for the negative evaluations, but on the other end of the spectrum. Most negative comments concerned women’s experiences of being mistreated or not taken seriously by the midwives or other care providers. Some women described absent midwives and not being seen, listened to, believed, or respected, resulting in the experience of not receiving proper support or adequate information, or not having sufficient pain relief. Ultimately, not building satisfactory rapport with the midwife greatly affected the women’s experience, leaving them with feelings of neglect and at times fear.

*‘There was one person who made me very nervous, she ‘forgot’ (I guess) about me several times and didn’t want to answer my questions but thought that we could discuss them later – even though I had written in my birth plan that I needed to be informed to stay calm. It felt as if this person had a bad day and that I was in her way. When she got off her shift I was terrified, not knowing what to expect from the next midwife. However, the new midwife and all the others I met afterwards were wonderful.’*

Demonstrating the importance of acknowledgement and validation, however small, a simple greeting could be the difference between a positive and a negative evaluation. In a caregiver-patient context, where one is the presumptive expert on the other’s bodily processes, common courtesy and mutuality becomes all the more important, and reduces the risk of perceived power imbalances and negative dependency.

*‘The doctor didn’t say ‘hello’ to me when he came into the delivery room the first time. He just talked to the midwife about the foetal monitoring. Some people should learn to treat people better.’*

For a woman to be able to make informed decisions regarding her own body during labour and birth, she needs to be given timely and adequate information about the progress of birth and of any intended intervention, so that she can make decisions that are right for her. Women who felt that they did not receive enough information or were included in decision-making expressed this in their evaluations. However, there were also examples of women showing great patience in not receiving sufficient information before different interventions, here before a caesarean section:

*‘I would have wanted a little more information before the surgery, but I know that it was a stressful situation with an overcrowded ward.’*

There were several comments about the midwife being under heavy workload, consequently absent from the birthing room, unable to provide sufficient support to the women/couple. Many of the negative evaluations demonstrate a busy labour ward with midwives going out of their way trying to provide the best care possible under stressful and demanding circumstances.

*‘As labour progressed so fast I really would have appreciated to have someone by my side, for example when I had a contraction that lasted 20 minutes. […] The county council should hire more midwives and raise the salaries. I was treated well when they had time for me.’*

In addition to comments concerning the negative consequences of low staffing, women also wrote about other implications of the organisational design of labour care. Some women described how shift work could have a negative impact on interpersonal communication, with the potential of information getting lost between midwives during the shift report, to the detriment of the birthing woman.

*‘We were a bit unlucky coming to the hospital in the middle of the change of shift and that everything went so fast that we didn’t know what happened. There was lots of miscommunication because of labour progressing at a fast rate and a late change of midwives. This could have been handled better but at the same time we understand that working hours are important. I really didn’t hear or understand whom to listen to.’*

A few women questioned some of the hospital routines and thought that the postpartum transfer between the delivery ward and the postnatal ward (on another floor in the same building) took too long, causing unnecessary stress, and delaying the need to relax, rest, and bond with the new-born. Other women offered ideas of improvement of the physical environment, like a blanket in the cold admittance room, a mattress to sleep on or a comfortable armchair for the partner, or a better equipped labour room.

## Discussion

The three themes that we identified illustrate how the birthing woman, the midwife, and the setting are closely interwoven in the fabric of childbirth experiences. *Grateful women and nurturing midwives doing gender together* demonstrates how gender-normative behaviour may influence a positive birth experience when based on a reciprocal relationship. *Managing ambiguous feelings by sympathising with the midwife,* shows how women’s internalised sense of gender can make women belittle their negative experiences and refrain from delivering criticism. *The midwifery model of relational care impeded by the labour care organisation* describes how the way in which women are cared for during labour and birth is regulated by an organisation not always adapted to the benefit of birthing women, but built on gendered notions of birthing women’s and midwives’ gratitude and compliance.

Gilligan [25] described how girls and women are expected, according to societal norms, to act in a certain way because of their gender: to be relational, caring, polite, and selfless, something that she denotes being subjected to the ‘tyranny of nice and kind’. In a similar vein, but in the context of childbirth, Martin [29] showed how some women were concerned with performing gender norms while birthing, feeling that they must continue to be nice, kind, relational, and selfless, despite the physical demands of labour and childbirth. Our results point in the same direction, with women’s positive birth evaluations being focused mainly on the relationship with the midwife, and women describing midwives in ways that we have interpreted as expressions of normative femininity, with midwives enacting these norms and birthing women valuing these expressions. Also, the accounts that describe women’s gratitude and how some women expressed ‘being allowed’ by the midwife to behave in certain ways, may be indicative of how women have adapted gender-appropriate behaviour, being nice, polite, relational, and privileging others’ perspectives on their own bodily processes even above their own, as described by Martin [29].

The ambiguous evaluations capture the complexity of maternal satisfaction, women being both satisfied and dissatisfied at the same time. Explicit in these evaluations was an underlying theme regarding how women managed conflicting feelings by downplaying their own wants and needs, and mitigated their critiques by being understanding and sympathetic to midwife and her working conditions, possibly highly aware of the current problems with understaffing, stress, and burnout in Swedish maternity care [40, 41]. Most of these women experienced difficult feelings during labour but were nevertheless thankful for the support they received. While this may be interpreted as women acknowledging the importance of midwifery support, it may also be viewed as expressions of normative constructs about selflessness as a core attribute of femininity and motherhood, women being more concerned with the needs and wishes of others than with their own [42].

A woman-centred care model, which includes the midwifery model of care, is known to affect women’s birth evaluations positively. Such models are based on reciprocal and equal relationships between woman and midwife, where the woman will be assigned both choices and control over her situation [1, 3, 43]. Accordingly, women were critical of care when they felt the labour organisation did not meet their needs, which was made visible through women’s perceptions of labour ward staffing, shift work, routines, and the physical environment. The negative evaluations detail how the woman-midwife relationship was affected by a stressful work environment for the midwives, keeping them from being present in the birthing room, which in turn made the women feel that they were invalidated and neglected, that they did not experience adequate support or pain relief, or receive personalised information. This promotes a view of a labour care organisation not always designed to benefit either birthing women or midwives.

Weighing external societal expectations of a gender-appropriate behaviour together with women’s internalised sense of gender as expressed through their birth evaluations, we contend that the birthing room is in no way excluded from gendered norms. However, adhering to or reinforcing ideal standards of femininity does not have to be negative per se, as the women in this study actually sought-after and appreciated these ideals when they perceived them in the midwives. Thus, it is of interest to discuss potential implications of women’s appreciation for and expressions of gender-normativity during childbirth, especially in a medicalised setting entailing various interventions on the woman’s body, known to influence maternal satisfaction negatively [1, 3, 11].

Among the women who partook in this study, nearly 8 out of 10 had some form of intrapartum intervention. Yet the women were generally very satisfied, which in part may be explained by the fact that people’s experiences are shaped by what they ‘know’, and that they tend to value the care they have experienced, remaining oblivious of other options [44]. Perhaps taking interventions during childbirth for granted, with some women expressing the desire for interventions, there were far more mentions of the midwife’s emotional support compared to her medical-technical skills, the latter encompassing various practices such as regular vaginal examination, intravenous and/or epidural infusion, amniotomy, continuous foetal monitoring, catheterisation, and episiotomy. This may in part be indicative that the midwives, despite a medicalised setting, had adopted a humanistic approach to care, merging technology with relationship-centred care – the so called ‘high tech, high touch’ approach [45, 46]. Although this approach aims to be inclusive and woman-centred, there are more issues with the approach to consider.

In the patriarchal, hierarchical, and medicalised care context of many Western labour care organisations, where the divide and power imbalances between caregiver and caretaker is more implicit and built into the system, there is a risk that the reciprocity of the woman-centred care model can be thwarted, with birthing women expected to maintain the role of the passive object [27, 47, 48, 49]. This could present a risk with the ‘high tech, high touch’ approach, as intrapartum interventions are intricately woven into labour care, partly through the institutional framing of birth as risk-laden and in need of ‘fixing’ [49]; and partly through the professional prerogative of the midwife, backed by the power of the institution [50]; and partly through the hands of a warm and kind midwife. We thus infer that in combination with women’s internalised sense of gender, the gendered social programming insisting that women be nice, kind, accommodating, patient and compliant, these factors may affect birthing women’s ability to assert themselves and be involved in decision-making during birth. Without underestimating the value of a warm and caring midwife, which was clearly appreciated by the majority of women in this study, we suggest that birthing women, through their internalised sense of gender are subjected to a certain degree to ‘the tyranny of nice and kind’, both in their views of the midwives and of their own behaviour.

For a deeper understanding of maternal satisfaction and the forces that shape women’s birth experiences, there is the need to not only consider institutional practices and discourses, but to also study the effects of women’s internalised sense of gender and expressions of normative femininity. To this end, we suggest further research in the form of ethnographic observations of the interaction between women and midwives during labour and birth.

### Methodological considerations

The large number of participants provided rich material, making word frequency and thematic analysis a suitable method of analysis for this data. Although we are aware that first-time mothers’ experiences may differ from those of women who had already given birth, we chose not to differentiate between the two groups in this study, since the aim was to describe variation in experiences rather than compare them.

Recognising the challenge of assigning the most value to the most commonly recurring words and thereby drawing conclusions, this method has nevertheless made our analysis very transparent, showing in detail the material that underpins the work, as suggested by Braun and Clarke [38]. The use of a gender perspective increased the level of analysis and deepened the discussion, thus strengthening our interpretations and providing an alternate way to view maternal satisfaction. To address trustworthiness according to Nowell et al. [37], researcher triangulation was used for enhanced credibility, involving all authors in the various steps of the analytic process. For transferability and confirmability, a description of the setting as well as quotes from the women’s evaluations were included and discussed. To achieve dependability, the research process has been clearly documented, including examples of coding and theme development.

More than half of the women in this study expressed exclusively positive comments about their birth experiences. Being careful not to undervalue the many positive evaluations, there is the possibility that the design of the evaluation form, as well as the fact that the form is a normal part of the labour ward’s quality management system, may have influenced the women’s responses. The form (i.e., the labour care quality administration) does not ask the women about negative experiences but about what was good and what could be better, thus implying that the women have little or nothing to be critical of, consequently risking avoiding potential negative criticism. Furthermore, the form is more focused on measuring staff performance and touches only superficially on the overall birth experience. Offering the women more examples of what to evaluate, such as continuity of care, physical environment, access to midwives, pain relief, birth tools, etc., may have provided more specific answers. During the course of the study, the limitations of the birth evaluation form became increasingly apparent, and in retrospect, creating another form may have added another dimension to the women’s evaluations. However, our findings may prompt the specific labour ward to revise the form to better capture women’s experiences.

There is also the aspect of the timing of the evaluation, as the initial relief of having a healthy baby may colour the first assessment of the birth experience, and the overall birth experience tends to become more negative over time, especially if it involved negative interactions with the staff [2, 6]. The women in this study were asked to evaluate their experiences within approximately 48 h after birth, and as a negative birth experience takes longer to integrate – women with a traumatic birth experience may still be in shock two days after birth, the evaluations may have been more negative had the women had more time to think through the experience [6]. Apart from the timing of the evaluation, there is also the need to consider that women, filling in the evaluation form while still at the hospital, because of gendered norms and expectations on women to be forgiving and grateful, may be hesitant to criticise their caregivers, leading to socially desirable responses or ingratiating response bias [44].

## Conclusion

Present work explores women’s evaluations of their birth experiences, and our findings reveal that most women were very satisfied, predominantly with the emotional support they received from the midwives; some had ambiguous feelings, being satisfied and dissatisfied at the same time; and some women were dissatisfied, mainly with the labour care organisation. The use of a gender perspective to underpin the study, and the performance of a latent and constructionist thematic analysis based on word count, enabled a deepened discussion of the women’s evaluations as reflections of the underlying sociocultural ideas, assumptions, and ideologies that shape women’s realities.

The women were found to value a midwife displaying traits associated with normative femininity according to Western standards, i.e., someone relationship-oriented, responsive, kind, and supportive. Through our interpretations, the women themselves also showed examples of a gender-normative behaviour, being thankful, sympathetic, and belittling of their own feelings or requests, despite the perceptions of some women that they did not have the support or birth experience they had hoped for. We also found examples of how the midwifery model of relational care was impeded by the labour care organisation, through inadequate staffing, shift work, routines, and physical environment, to the detriment of the birthing woman.

In addition to elucidating the impact of institutional, i.e., medical, practices and discourses on childbirth, there is also the need to consider how birthing women’s internalised sense of gender, i.e., the way individuals behave, and are expected to behave, according to societal and cultural norms, interplay with and affect their birth experiences. A gender perspective may provide a useful tool in unveiling gender-normative complexities surrounding the childbirth experience.

## Data Availability

The datasets generated and/or analysed during the current study are not publicly available due to their containing information that could compromise the privacy of research participants, but are available from the corresponding author on reasonable request.

## Abbreviation

VAS: Visual Analogue Scale
